# The olfactory receptor OR51E2 activates ERK1/2 through the Golgi-localized Gβγ-PI3Kγ-ARF1 pathway in prostate cancer cells

**DOI:** 10.3389/fphar.2022.1009380

**Published:** 2022-10-13

**Authors:** Xin Xu, Mostafa Khater, Guangyu Wu

**Affiliations:** Department of Pharmacology and Toxicology, Medical College of Georgia, Augusta University, Augusta, GA, United States

**Keywords:** G protein-coupled receptor, olfactory receptor OR51E2, Gβγ, PI3Kγ, ARF1, Golgi translocation, ERK1/2, prostate cancer

## Abstract

The olfactory receptor OR51E2 is ectopically expressed in prostate tissues and regulates prostate cancer progression, but its function and regulation in oncogenic mitogen-activate protein kinase (MAPK) activation are poorly defined. Here we demonstrate that β-ionone, an OR51E2 agonist, dose-dependently activates extracellular signal-regulated kinases 1 and 2 (ERK1/2) in prostate cancer cells, with an EC50 value of approximate 20 μM and an efficiency comparable to other receptor agonists. We also find that CRISPR-Cas9-mediated knockout of Golgi-translocating Gγ9 subunit, phosphoinositide 3-kinase γ (PI3Kγ) and the small GTPase ADP-ribosylation factor 1 (ARF1), as well as pharmacological inhibition of Gβγ, PI3Kγ and Golgi-localized ARF1, each abolishes ERK1/2 activation by β-ionone. We further show that β-ionone significantly promotes ARF1 translocation to the Golgi and activates ARF1 that can be inhibited by Gγ9 and PI3Kγ depletion. Collectively, our data demonstrate that OR51E2 activates ERK1/2 through the Gβγ-PI3Kγ-ARF1 pathway that occurs spatially at the Golgi, and also provide important insights into MAPK hyper-activation in prostate cancer.

## Introduction

Olfactory receptors (ORs) are specific G protein-coupled receptors (GPCRs) that are responsible for the detection of odor molecules to control the sense of smell. Although ORs were first described to be exclusively expressed in the olfactory epithelium of chemosensory neurons, some ORs have been found in various non-olfactory tissues. One of such ORs is OR51E2 which is highly expressed in human prostate tissues and thus, it is known as a prostate-specific GPCR (Xia et al., 2001; Wang et al., 2006; Xu et al., 2006). OR51E2 also exists in airway smooth muscle cells (Aisenberg et al., 2016), melanocytes (Gelis et al., 2016) and retinal pigment epithelial cells (Jovancevic et al., 2017). The most studies on OR51E2 have been focused on its pathophysiological functions and these studies have demonstrated that it regulates prostate cancer cell proliferation, invasion and migration, and prostate cancer progression and can be used as a prostate cancer biomarker (Weng et al., 2005; Neuhaus et al., 2009; Rigau et al., 2010; Spehr et al., 2011; Rodriguez et al., 2014; Sanz et al., 2014; Rodriguez et al., 2016; Sanz et al., 2017a; Sanz et al., 2017b; Xie et al., 2019; Pronin and Slepak, 2021). At the molecular level, OR51E2 was shown to interact with G protein Gα12 (Xia et al., 2001) which led to the identification of the receptor and enhance Ca^2+^ signal, cAMP production and protein kinase activation (Neuhaus et al., 2009; Spehr et al., 2011; Gelis et al., 2016; Jovancevic et al., 2017; Pronin and Slepak, 2021). Recent studies suggest that OR51E2 activates mitogen-activated protein kinases (MAPKs), including extracellular signal-regulated protein kinases 1 and 2 (ERK1/2) (Jovancevic et al., 2017; Pronin and Slepak, 2021), with the detailed mechanisms being undefined.

The MAPK Raf-MEK-ERK1/2 pathway plays a crucial role in many fundamental cellular processes and its hyper-activation is directly associated with the pathogenesis of human diseases, particularly cancer. Studies in the past decades have identified a number of genetic mutations in the signaling molecules involved in this MAPK pathway, including receptor tyrosine kinases (RTKs), Ras, Raf, and MEK. These mutations constitutively activate the MAPK pathway to drive many types of malignancies for which Raf and MEK inhibitors have been developed to treat the diseases. Similar to many other cancer types, the MAPK pathway is highly activated in prostate cancer, and importantly the hyper-activation of this pathway is correlated with prostate cancer progression, androgen independence and poor prognosis (Gioeli et al., 1999; Uzgare et al., 2003; Weber and Gioeli, 2004; Roberts and Der, 2007; Imada et al., 2013). As such, the signaling molecules involved in the regulation of this pathway have been thought to be suitable targets for therapeutic intervention and extensive efforts have been made to identify the factors that control the MAPK activation in prostate cancer cells. However, prostate cancer patients do not frequently carry abovementioned oncogenic mutations. Despite the fact that downregulation of several MAPK negative regulators, such as the Sprouty family members and Raf kinase inhibitor protein (RKIP), may enhance MAPK activation in prostate cancer (Schutzman and Martin, 2012), the molecular mechanisms underlying the hyper-activation of the MAPK pathway in prostate cancer remain elusive.

Heterotrimeric G proteins, consisting of α, β, and γ subunits, are the major signaling mediators of GPCRs. Once activated by GPCRs, Gα subunits will dissociate from Gβγ dimers and then, both Gα and Gβγ can separately interact with and modulate the activity of specific downstream effectors (Wu et al., 1998; Wu et al., 2000; Khan et al., 2013). It is well known that GPCRs can activate the MAPK pathway through multiple signaling molecules, including Gβγ complex, and Gβγ-initiated signaling event is generally considered to occur at the plasma membrane (PM) (Crespo et al., 1994; Koch et al., 1994; Khan et al., 2013). Recent studies have demonstrated that GPCR activation at the PM induces the translocation of some Gβγ dimers from the PM to the Golgi apparatus (GA) and that the translocation efficiency is determined by Gγ subunits (Akgoz et al., 2004; Akgoz et al., 2006; Saini et al., 2007; Chisari et al., 2009; O'Neill et al., 2012; Senarath et al., 2018; Khater et al., 2021a; Khater et al., 2021b). The Gβγ complex at the GA can activate phospholipase C (Malik et al., 2015; Madukwe et al., 2018) and protein kinase D (Jamora et al., 1999; Irannejad and Wedegaertner, 2010) and regulate post-Golgi trafficking (Irannejad and Wedegaertner, 2010; Jensen et al., 2016; Klayman and Wedegaertner, 2017), Golgi structure and fragmentation (Jamora et al., 1999; Saini et al., 2010; Klayman and Wedegaertner, 2017; Rajanala et al., 2021), insulin secretion (Saini et al., 2010), and cardiomyocyte hypertrophic growth (Malik et al., 2015). We have recently identified a novel function for Gβγ translocation to the GA to activate the MAPKs ERK1/2 in prostate cancer cells and this function is mediated through phosphoinositide 3-kinase γ (PI3Kγ), a well-characterized Gβγ downstream effector, and the small GTPase ADP-ribosylation factor 1 (ARF1) (Khater et al., 2021a; Khater et al., 2021b). The purposes of this study are to characterize the function of ectopically expressed OR51E2 in ERK1/2 activation and to elucidate the possible underlying molecular mechanisms in prostate cancer cells.

## Materials and methods

### Materials

Mouse monoclonal anti-FLAG M2 antibodies (F-3165), UK14304 (U104), isoproterenol (Iso, I2760), angiotensin II (Ang II, 05-23-0101) adenosine (A4036), endothelin (E7764), oxotremorine M (Oxo-M, O100), sphingosine-1-phosphate (S1P, S9666), Exo2 (E7159) and 5-hydroxytryptamine (5HT) were purchased from Sigma-Aldrich. Rabbit polyclonal anti-ERK1/2 antibodies (sc-7383), secinH3 (sc-203260) and golgicide A (GCA, sc-215103) were from Santa Cruz Biotechnology. Lipopolysaccharide (LPS, 00-4976-93), β-ionone (297130050), lipofectamine 3000 (L3000-015), goat anti-mouse IgG (H+L) Alexa Fluor 488 (A-11001), goat anti-rabbit IgG (H+L) Alexa Fluor 594 (A-11012) and goat anti-mouse IgG (H+L) Alexa Fluor 594 (A-11032), were from Thermo Fisher Scientific. Rabbit polyclonal anti-ARF1 antibodies (ab183576) were from Abcam. Mouse anti-human p230 antibodies (611280) were from BD Biosciences. Rabbit polyclonal anti-ERK1/2 antibodies (9102) were from Cell Signaling Technology. Gallein (3090) was from Tocris Bioscience. AS-604850 (B2181) was from ApexBio.

### Plasmid construction

FLAG-tagged OR51E2 was kindly provided by Dr. Jennifer L. Pluznick as described (Shepard et al., 2013; Aisenberg et al., 2016). GFP-tagged ARF1 mutants were generated using the BamHI and EcoRI restriction sites of the pEGFP-N1 vector as described previously (Dong et al., 2011). ARF1 mutants were generated by QuikChange site-directed mutagenesis. All constructs used in the present study were verified by nucleotide sequence analysis.

### Cell culture

All cells were purchased from American Type Culture Collection (ATCC, Rockville, MD). Prostate cancer DU145 and LNCaP cells were cultured in complete Roswell Park Memorial Institute (RPMI) 1640 medium supplemented with 2 mM L-glutamine and 10% fetal bovine serum (FBS). HEK293 cells were cultured in Dulbecco’s modified Eagle’s medium (DMEM) with 10% FBS.

### Transient transfection

For analysis of the subcellular distribution of OR51E2 and measurement of ERK1/2 activation by OR51E2 in HEK293 cells, the cells were cultured in 6-well dishes and transfected with FLAG-tagged OR51E2 (1 μg) for 24 h using Lipofectamine 3000. Similarly, the cells were transfected with GFP-tagged ARF1Q71L or ARF1T31N for analysis of the subcellular distribution of ARF1.

### Generation of knockout (KO) cell lines using the CRISPR-Cas9 genome editing technology

Gγ9, Gγ3, and p110γ KO cells were generated by using the CRISPR-Cas9 system as described previously (Khater et al., 2021b). Briefly, sgRNAs targeting Gγ9, Gγ3 and p110γ were constructed into the lentiCRISPR v2 vector (Addgene plasmid #52961). The plasmids containing sgRNAs were transfected into cells using Lipofectamine 3000 and the cells were selected in puromycin at a concentration of 10 μg/ml. KO of the targeted proteins were determined by Western blotting.

### KO of ARF1 by CRISPR-Cas9 KO plasmids

ARF1 KO was achieved by transient transfection of CRISPR-Cas9 KO plasmids as described (Khater et al., 2021a; Wei et al., 2021). CRISPR-Cas9 KO plasmids targeting human ARF1, as well as control plasmids, were purchased from Santa Cruz Biotechnology. The KO plasmid consists of a pool of three plasmids, each encoding the Cas9 nuclease and a target-specific 20 sgRNA. Cells were cultured on 6-well plates and transfected with KO plasmids (1 μg) using Lipofectamine 3000 for 24 h. The cells were transfected again for another 24 h. The cells were split at a ratio of 1:2, grown for additional 24 h and then starved for 48 h before stimulation with β-ionone at 100 μM for 5 min.

### Measurement of ERK1/2 activation

Cells were cultured in 6-well dishes for 24 h and starved for 24 h (HEK293 cells) or 48 h (prostate cancer cells) before stimulation with β-ionone or other agonists as indicated in the figure legends. After the medium was removed and the cells were washed twice with cold phosphate-buffered saline (PBS), the cells were solubilized by the addition of 300 μl of 1X SDS gel-loading buffer. ERK1/2 activation was determined by measuring ERK1/2 phosphorylation by Western blotting as described previously (Wu et al., 2003). ERK1/2 activation was calculated either by percentages relative to the maximal response or by fold increase over the basal level.

### GST fusion protein pulldown assays

ARF1 activation was measured in GST fusion protein pulldown assays using the GGA3 VHS-GAT domains which specifically bind the active form of ARF1 as described (Dell'Angelica et al., 2000; Zhou et al., 2015). GST fusion proteins were purified by using MagneGST™ glutathione purification system (Promega) and analyzed by Coomassie Brilliant blue staining following SDS-PAGE before experiments. To measure ARF1 activation, DU145 cells were cultured on 12-well dishes for 24 h and starved for 48 h. The cells were then stimulated for β-ionone at 100 μM for 5 min. After washing with cold PBS twice, the cells were lysed with buffer containing 50 mM Tris-HCl, pH 7.4, 10 mM MgCl_2_, 300 mM NaCl, 2% Nonidet P-40, 0.01% SDS and 1 X protease inhibitor cocktail (Roche). After sonication, total cell lysates were centrifuged at 100,000 X rpm for 20 min at 4°C, and the supernatants were incubated with glutathione beads with gentle rotation at 4°C overnight. The beads were washed three times with buffer containing 25 mM Tris-HCl, pH 7.4, 30 mM MgCl_2_, 150 mM NaCl, and 1% Nonidet P-40. Active ARF1 bound to the beads was eluted with 2X SDS-gel loading buffer and detected by immunoblotting using ARF1 antibodies.

### Fluorescence microscopy

For analysis of subcellular distribution of OR51E2, HEK293 cells were cultured on coverslip precoated with poly-L-lysine on 6-well dishes and transiently transfected with 1 μg of FLAG-tagged OR51E2 for 24 h. The cells were fixed and permeabilized with PBS containing 0.2% Triton X-100 for 5 min. After blocking with 0.24% normal donkey serum for 1 h, the cells were stained with primary antibodies against FLAG (1:50 dilution) overnight followed by staining with AlexFluor-conjugated secondary antibodies for 1 h. To study the subcellular localization of ARF1 mutants, GFP-tagged ARF1Q71L or ARF1T31N was transiently expressed. To study the translocation of endogenous ARF1 in prostate cancer cells, the cells were cultured on coverslip, starved for 48 h and then stimulated with β-ionone at 100 μM for 5 min. After the cells were fixed, permeabilized and blocked as above, the cells were stained with primary antibodies against ARF1 and p230 (1:50 dilution) overnight followed by staining with AlexFluor-conjugated secondary antibodies. All images were captured with a ×63 objective on a Leica Stellaris 5 confocal microscope as described previously (Wei et al., 2019; Xu et al., 2022). Total ARF1 expression and ARF1 expression at the GA were quantified by NIH ImageJ using p230 as a Golgi marker as described previously (Xu et al., 2022).

### Statistical analysis

Statistical analysis was performed using unpaired Student’s t test. P< 0.05 was considered as statistically significant. All data were presented as mean ± SE.

## Results

### OR51E2 activates ERK1/2 in prostate cancer cells

**FIGURE 1:**
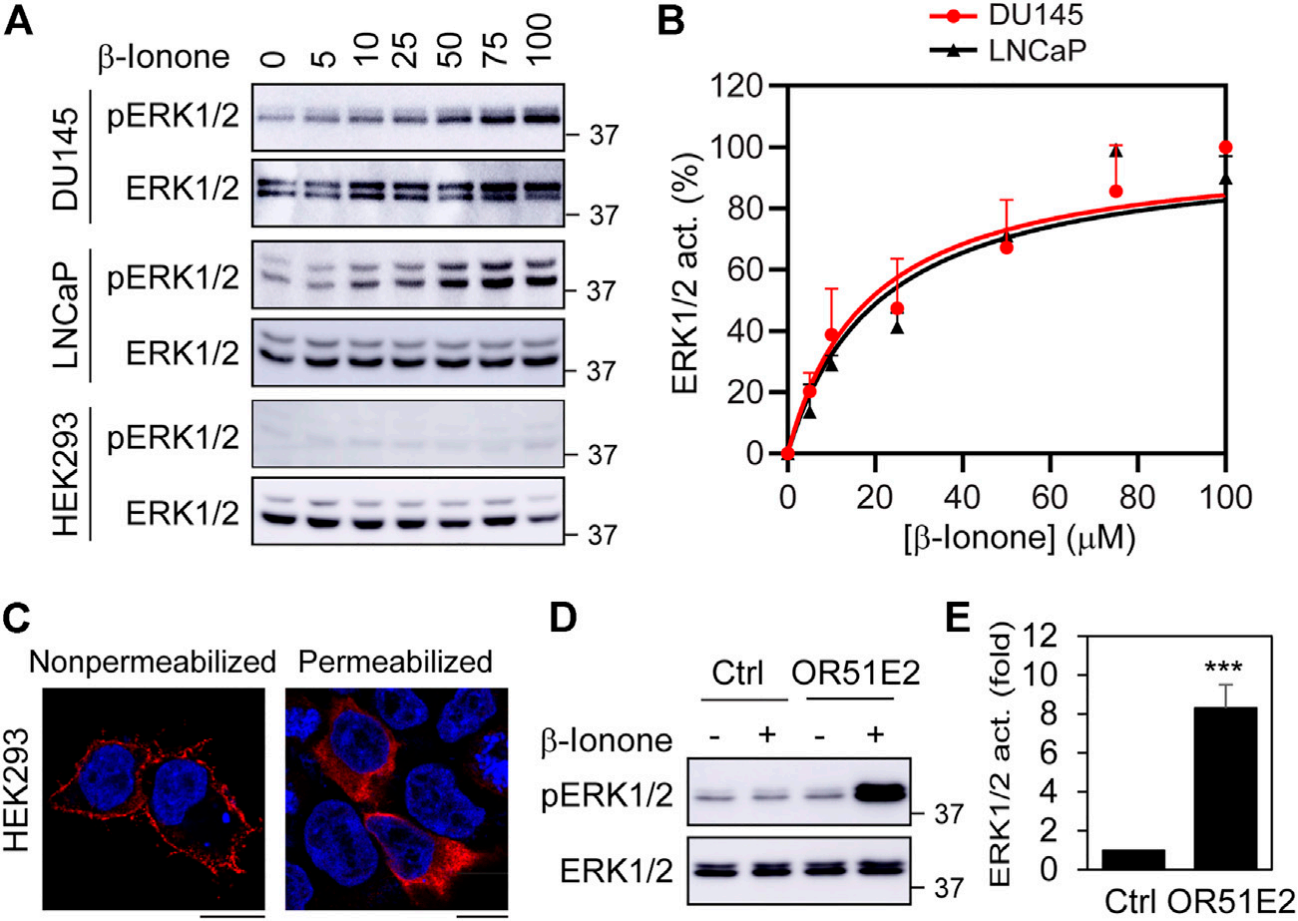
ERK1/2 activation by ectopically expressed OR51E2 in DU145, LNCaP and HEK293 cells. **(A)** ERK1/2 activation by β-Ionone. The cells were cultured on 6-well dishes, starved for 24 h (HEK293 cells) or 48 h (DU145 and LNCaP cells), and then stimulated with different concentrations of β-ionone for 5 min. **(B)** Quantitative data shown in A. **(C)** Subcellular distribution of FLAG-OR51E2 in HEK293 cells revealed by confocal microscopy. HEK293 cells were transfected with FLAG-tagged OR51E2 for 24 h and stained with FLAG antibodies with (right panel) or without (left panel) permeablization with Triton X-100 for 5 min. The images shown are representatives of three experiments. Scale bars: 10 μm. **(D)** ERK1/2 activation by β-ionone in HEK293 cells expressing exogenous OR51E2. HEK293 cells were transfected with OR51E2 for 24 h and stimulated with β-ionone at 100 μM for 5 min after starvation for 24 h. **(E)** Quantitative data shown in D. The quantitative data are presented as means ± SE (*n* = 3). ***p < 0.001 versus control.

As an initial approach to characterize ERK1/2 activation by OR51E2, we determined the effect of stimulation with increasing concentrations of β-ionone, a newly identified OR51E2 agonist (Neuhaus et al., 2009), in two prostate cancer cell lines (DU145 and LNCaP). HEK293 cell line, which does not express endogenous OR51E2, was used as a negative control. Stimulation with β-ionone activated ERK1/2 in a dose-dependent fashion in both prostate cancer cells with an EC50 value of approximate 20 μM (Figures 1A,B). In contrast, β-ionone stimulation had no effect on ERK1/2 activation in HEK293 cells. These data demonstrate that activation of endogenous OR51E2 by β-ionone is able to activate the MAPKs ERK1/2 in prostate cancer cells.

We then determined if transient expression of FLAG-tagged OR51E2 could activate ERK1/2 in HEK293 cells. Although ORs are efficiently expressed at the cell surface in olfactory neurons, they are retained in intracellular compartments (e.g., ER, GA, and endosomes) when expressed in heterologous systems (Shepard et al., 2013). Confocal microscopy showed that FLAG-tagged OR51E2 was clearly detected at the cell surface after staining with anti-FLAG antibodies in non-permeabilized cells, but a significant amount of the receptors was found inside the cell in permeabilized cells (Figure 1C). β-Ionone stimulation robustly activated ERK1/2 in HEK293 cells expressing FLAG-OR51E2 (Figures 1D,E), suggesting that, similar to endogenous OR51E2 in prostate cancer cells, exogenous FLAG-OR51E2 is fully capable of activating ERK1/2 in HEK293 cells.

**FIGURE 2:**
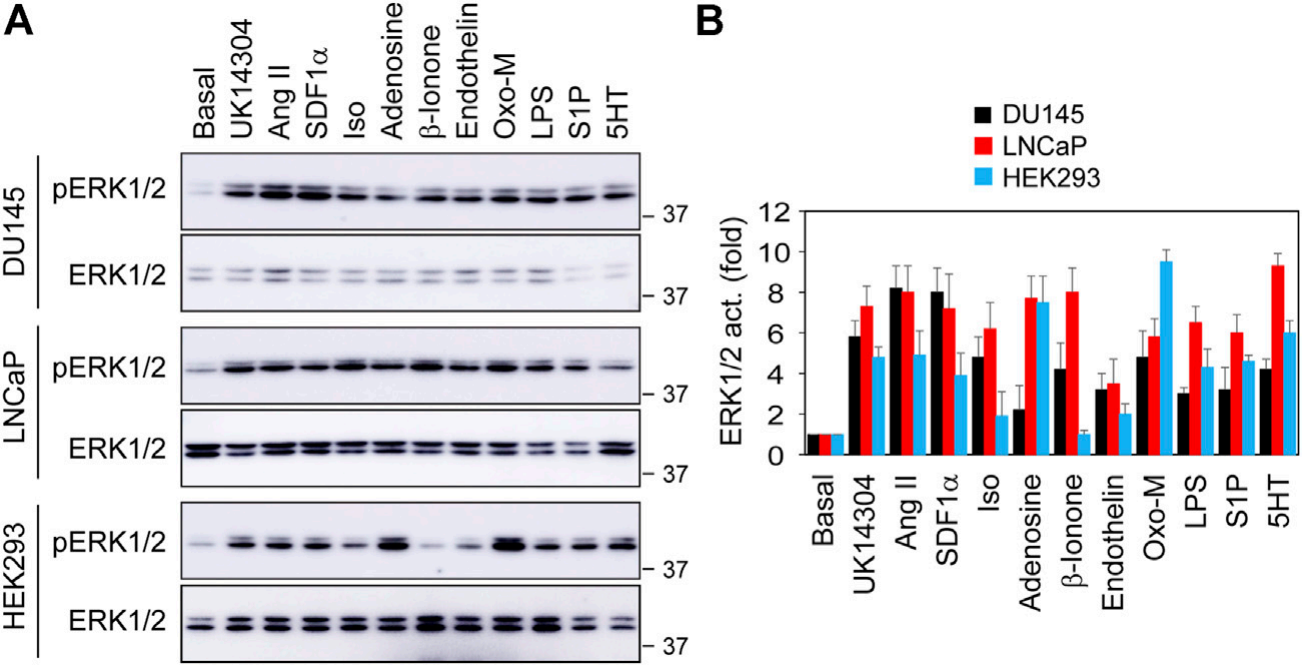
Comparison of ERK1/2 activation by different GPCR agonists in DU145, LNCaP and HEK293 cells. **(A)** ERK1/2 activation in response to stimulation with different GPCR agonists and LPS. The cells were cultured on 6-well dishes, starved for 24 h (HEK293 cells) or 48 h (DU145 and LNCaP cells) and then stimulated for 5 min with UK14340 (1 μM), Ang II (1 μM), SDF1α (25 nM), Iso (10 μM), β-ionone (100 μM), adenosine (10 μM), endothelin (100 nM), Oxo-M (10 μM), S1P (125 nM), 5HT (15 nM), and LPS (1 μg/ml). **(B)** Quantitative data shown in A. The quantitative data are presented as means ± SE (*n* = 3).

We next compared OR51E2’s ability to activate ERK1/2 with other GPCRs. For this purpose, we measured ERK1/2 activation in DU145, LNCaP and HEK293 cells by ten GPCR agonists, including UK14340 for α2-adrenergic receptors (α2-ARs), Ang II for Ang II receptors, Iso for β2-AR, adenosine for adenosine receptors, β-ionone for OR51E2, endothelin for endothelin receptors, Oxo-M for muscarinic receptors, S1P for S1P receptors, and 5HT for 5HT receptors. In addition, LPS, which is well known to strongly activate the MAPK pathway, was also used. Although the activation magnitudes were variable, all ten GPCR agonists and LPS significantly activated ERK1/2 in 3 cell lines, except that β-ionone was unable to activate ERK1/2 in HEK293 cells (Figures 2A,B). ERK1/2 activation by β-ionone was comparable to other nine GPCR agonists tested in prostate cancer cells. These data suggest that, similar to many other GPCRs, OR51E2 is a potent activator of ERK1/2 in prostate cancer cells.

### Pharmacological inhibition of Gβγ and PI3Kγ and CRISPR-Cas9-mediated KO of Gγ9 and p110γ abolish ERK1/2 activation by OR51E2

**FIGURE 3:**
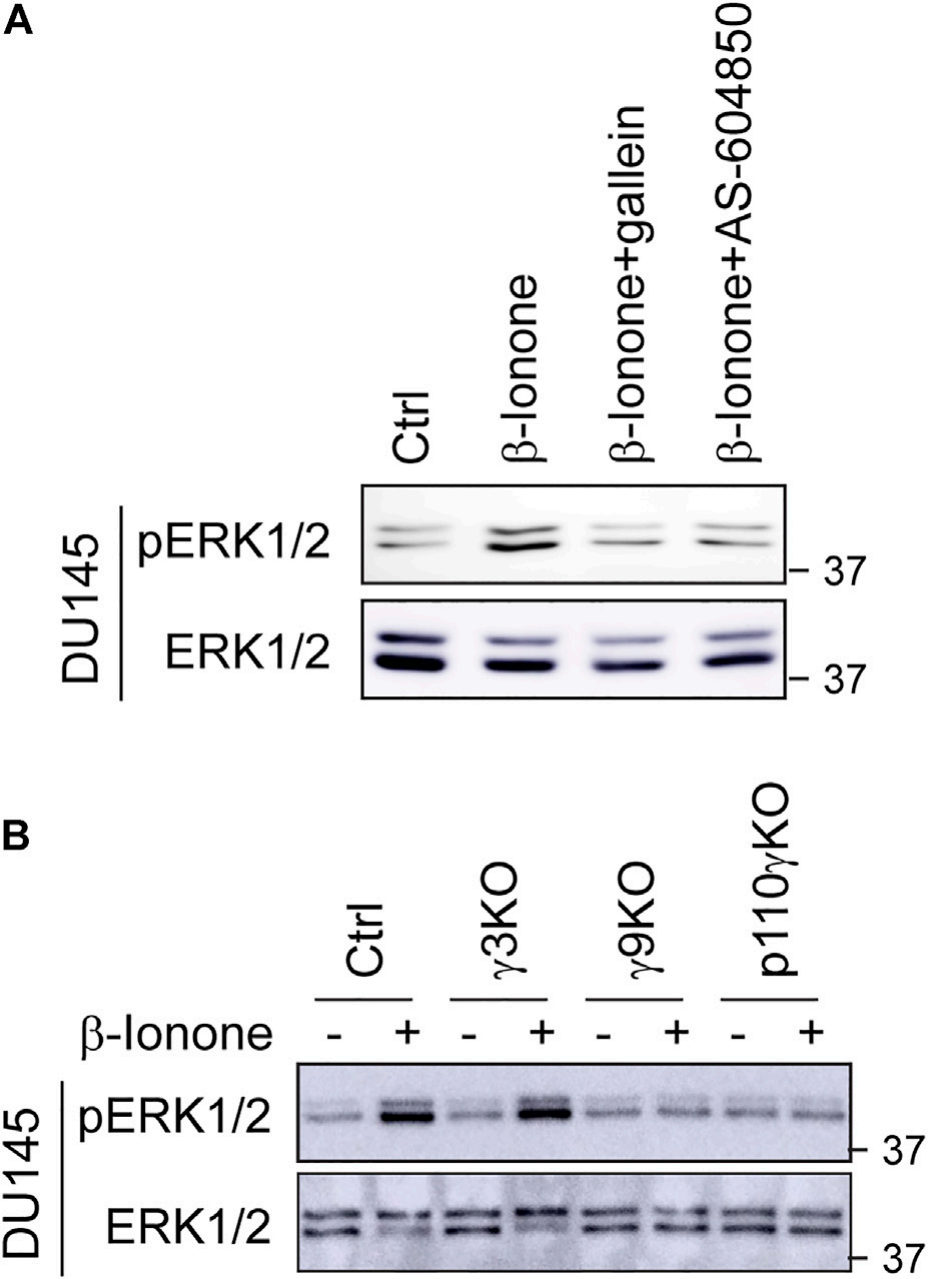
Pharmacological inhibition of Gβγ and PI3Kγ and CSRIPR-Cas9-mediated KO of Gγ9 and p110γ suppress ERK1/2 activation by OR51E2. **(A)** Inhibition of β-ionone-mediated ERK1/2 activation by Gβγ and PI3Kγ inhibitors. DU145 cells were starved for 48 h and then treated with gallein (10 μM for 30 min) or AS-604850 (2.5 μM for 6 h) before stimulation with β-ionone at 100 μM for 5 min **(B)** ERK1/2 activation in Gγ3, Gγ9 and p110γ KO cells in response to β-ionone stimulation. DU145 KO cells were starved for 48 h before stimulation with β-ionone. The Western blots shown are representatives of at least three experiments.

We have recently demonstrated that, among 12 Gγ subunits, Gγ9 is the most Golgi-translocating Gγ subunit, whereas Gγ3 is the least GA-translocating Gγ subunit in prostate cancer cells and that the chemokine receptor CXCR4 activates ERK1/2 through a novel pathway involving Gβγ translocation to the GA and PI3Kγ activation (Khater et al., 2021b). To study the molecular mechanisms underlying ERK1/2 activation by OR51E2, we first determined the effect of pharmacological inhibition of Gβγ and PI3Kγ on ERK1/2 activation by OR51E2. Treatment with the specific Gβγ inhibitor gallein and the specific PI3Kγ inhibitor AS-604850 dramatically attenuated ERK1/2 activation by β-ionone in DU145 cells (Figure 3A), suggestive of a role of Gβγ and PI3Kγ in OR51E2-mediated ERK1/2 activation in prostate cancer calls.

We then took advantage of previously generated cell lines in which Gγ9, Gγ3, and the PI3Kγ catalytic subunit p110 (p110γ) were individually depleted by CRISPR-Cas9 genome editing and measured ERK1/2 activation in these KO cells. KO of Gγ9, but not Gγ3, markedly inhibited ERK1/2 activation after β-ionone stimulation in DU145 cells (Figure 3B). p110γ KO also strongly suppressed ERK1/2 activation by β-ionone (Figure 3B). These data suggest that Gβγ translocation to the GA is a crucial event in ERK1/2 activation by OR51E2.

### OR51E2 induces the GA translocation and activation of ARF1 *via* Gβγ and PI3Kγ in prostate cancer cells

**FIGURE 4:**
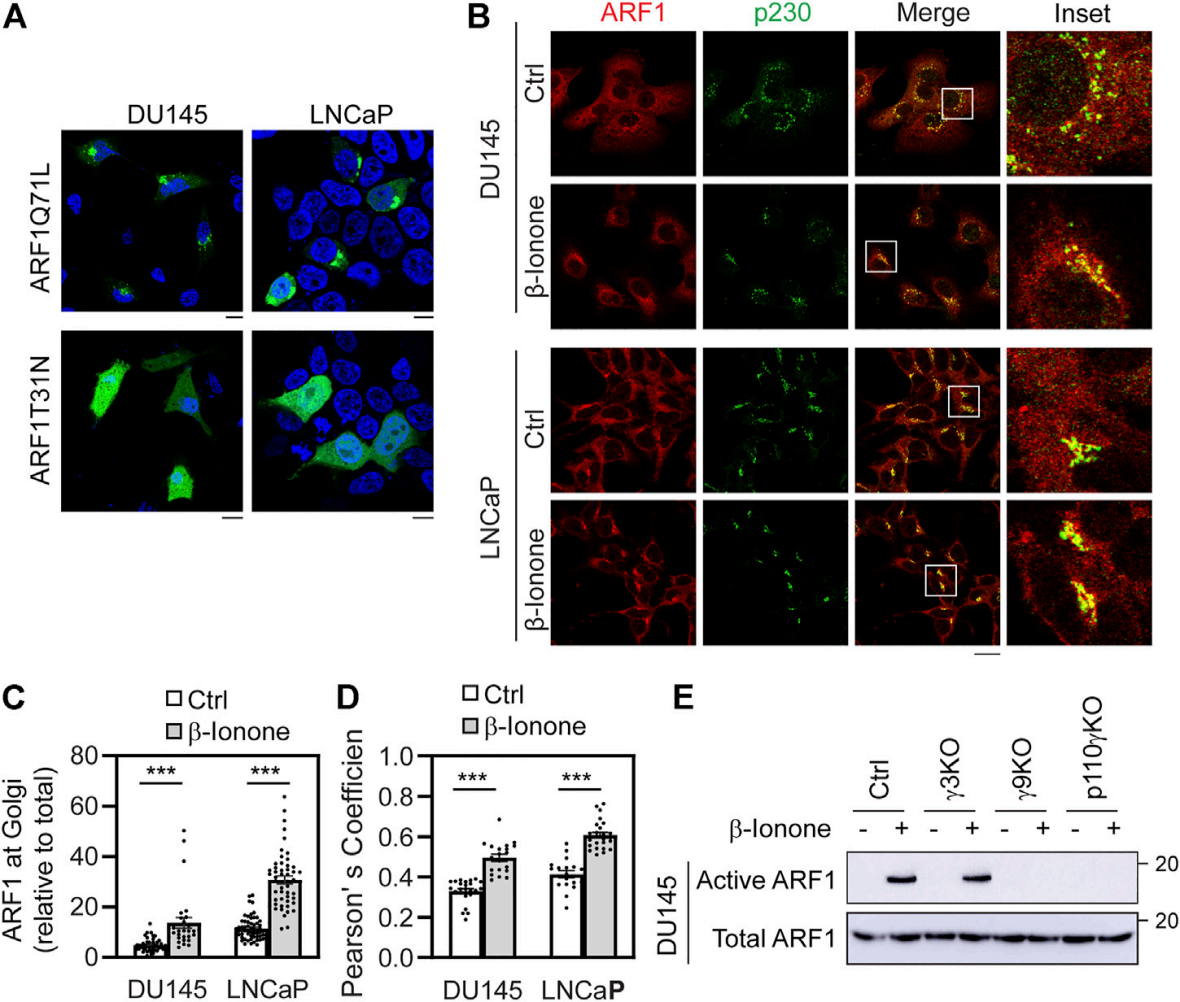
Stimulation with β-ionone recruits ARF1 to the GA and activates ARF1 *via* Gβγ and PI3Kγ in prostate cancer cells. **(A)** Subcellular distribution of ARF1Q71L and ARF1T31N in DU145 and LNCaP cells. The cells were transfected with GFP-tagged ARF1 active or dominant negative mutant for 24 h and the subcellular distribution of ARF1 mutants was revealed by confocal microscopy. **(B)** ARF1 translocation to the GA after β-ionone stimulation. The cells were starved for 48 h, stimulated with β-ionone at 100 μM for 5 min and then stained with antibodies against ARF1 and p230. **(C)** Quantification of ARF1 expression at the GA by using p230 as a GA marker. **(D)** Quantification of Pearson’s coefficient between ARF1 and p230. **(E)** ARF1 activation in Gγ3, Gγ9, and p110γ KO cells in response to β-ionone stimulation. DU145 KO cells were starved for 48 h before stimulation with β-ionone at 100 μM for 5 min. ARF1 activation was measured in GST fusion protein pulldown assays. The quantitative data are presented as means ± SE (*n* = 29–43 cells in C and 18–25 cells in D in three individual experiments). ***, *p* < 0.001 *versus* respective control. Scale bar: 10 μm. The Western blots shown are representatives of three experiments.

ARF1 is a Ras-like small GTPase which is best known for its functions in maintaining the structure and function of the GA and in vesicular trafficking, particularly in the formation of COPI- and clathrin-coated vesicles which mediate cargo transport between the ER and the GA and between the TGN and endosomes, respectively (Donaldson and Jackson, 2011). It has been demonstrated that membrane-associated ARF1 is active form, whereas its cytosolic form is inactive. Indeed, the constitutively active GTP-bound mutant ARR1Q71L mainly localized at the GA, whereas the dominant-negative GDP-bound mutant ARF1T31N largely localized in cytoplasm in prostate cancer DU145 and LNCaP cells (Figure 4A).

Previous studies have demonstrated that GPCRs can interact with and activate ARF1 and ARF1 regulates GPCR biosynthesis and signaling (Mitchell et al., 1998; Mitchell et al., 2003; Dong et al., 2010; Dong et al., 2011). Transient expression of ARF1 or ARF1Q71L induces robust activation of ERK1/2 (Dong et al., 2011; Zhou et al., 2015; Davis et al., 2016). We have recently shown that, in addition to Gβγ and PI3Kγ, ARF1 activation also plays an essential role in ERK1/2 activation by CXCR4 in prostate cancer cells (Khater et al., 2021a). To determine the effect of OR51E2 on ARF1 activation, we first defined if OR51E2 activation affected endogenous ARF1 translocation to the GA by confocal microscopy. After stimulation with β-ionone, ARF1 expression at the GA was augmented by 183% and 168% in DU145 and LNCaP cells, respectively, as quantified by using p230 as a GA marker (Figures 4B,C). Quantification of the colocalization of ARF1 with p230 using Pearson’s coefficient showed that β-ionone stimulation significantly enhanced ARF1 expression at the GA (Figure 4D).

We then directly measured ARF1 activation by OR51E2 in response to β-ionone stimulation and determined the effect of Gγ9, Gγ3, and p110γ KO on ARF1 activation in GST fusion protein pulldown assays using the VHS-GAT domains of GGA3 (Dell'Angelica et al., 2000; Zhou et al., 2015). Consistent with its effect on ARF1 translocation to the GA, β-ionone stimulation strongly potentiated ARF1 activation and this effect was completely reversed by depletion of Gγ9 and p110γ, whereas depletion of Gγ3 had no effect on ARF1 activation by β-ionone (Figure 4E). These data demonstrate that OR51E2 activates ARF1, likely *via* Gβγ translocation to the GA and PI3Kγ activation.

### KO of ARF1 and inhibition of its activation at the GA suppress ERK1/2 activation by OR51E2 in prostate cancer cells

**FIGURE 5:**
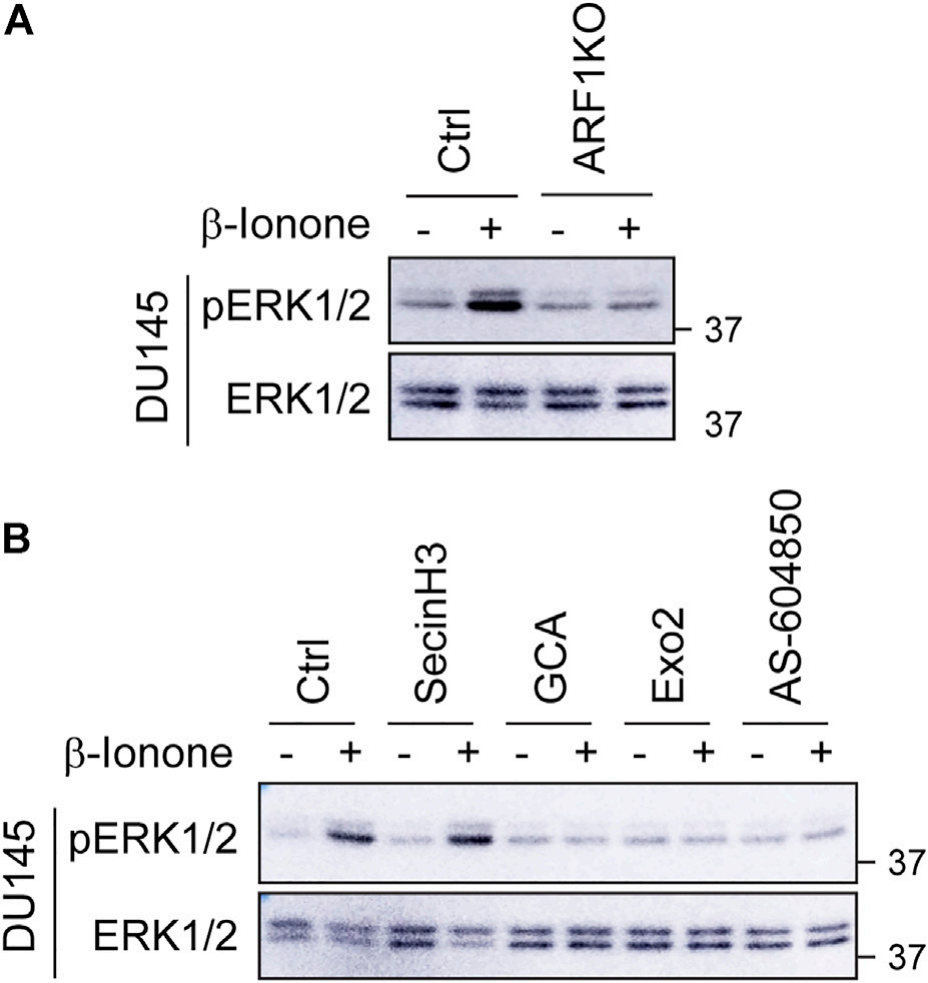
KO and inhibition of ARF1 abolish ERK1/2 activation by OR51E2. **(A)** Effect of CRISPR-Cas9-mediated depletion of ARF1 on ERK1/2 activation by β-ionone in DU145 cells. The cells were transiently transfected with CRISPR-Cas9 KO plasmids targeting human ARF1, starved for 48 h and then stimulated with β-ionone at 100 μM for 5 min. **(B)** Effect of ARF1 inhibitors on ERK1/2 activation by β-ionone in DU145 cells. The cells were starved for 48 h and treated with secinH3 (100 μM), GCA (30 μM) or Exo2 (60 μM) for 30 min before stimulation with β-ionone. AS-604850 treatment (2.5 μM for 6 h) was used as a positive control. The Western blots shown in each panel are representatives of at least three experiments.

To study the role of ARF1 in ERK1/2 activation by OR51E2 in prostate cancer cells, we determined the effect of CRISPR-Cas9-mediated KO of ARF1 *via* transient expression of CRISPR-Cas9 KO plasmids targeting ARF1 as described previously (Khater et al., 2021a) and specific pharmacological inhibition of ARF1 guanine nucleotide exchange factors (GEFs) at either the GA or the PM. Similar to the results observed in Gγ9 and PI3Kγ KO cells, expression of ARF1 KO plasmids blocked ERK1/2 activation by β-ionone stimulation in DU145 cells (Figure 5A). Treatment with golgicide A (GCA) and Exo2, two well-studied GA-localized ARF1GEF inhibitors dramatically attenuated ERK1/2 activation by β-ionone (Figure 5B). By contrast, treatment with secinH3, a PM-localized ARF1GEF inhibitor, did not affect ERK1/2 activation by β-ionone (Figure 5B). These data suggest that the activation of ARF1 at the GA, rather at the PM, mediates ERK1/2 activation by OR51E2 in prostate cancer cells.

## Discussion

In this study, we have demonstrated that ectopically expressed OR51E2 is a potent activator of the MAPKs ERK1/2 in prostate cancer cells. Stimulation with β-ionone dose-dependently activates ERK1/2 with an EC50 value of about 20 μM, and its efficiency is comparable to other GPCR agonists tested and LPS in two prostate cancer cell lines. As β-ionone stimulation does not activate ERK1/2 in HEK293 cells which lack endogenous OR51E2, but does cause robust ERK1/2 activation when exogenous OR51E2 is transiently expressed, OR51E2 activation by β-ionone, leading to ERK1/2 activation, is likely specific.

As the first study to elucidate the molecular mechanisms underlying ERK1/2 activation by OR51E2, we focus on three important signaling molecules: Gβγ, PI3Kγ and ARF1, which we have recently demonstrated to control CXCR4-mediated ERK1/2 activation in prostate cancer cells (Khater et al., 2021a; Khater et al., 2021b). There are several important points regarding the role of Gβγ, PI3Kγ and ARF1 in mediating ERK1/2 activation by OR51E2 in prostate cancer cells. 1) We have demonstrated that KO of Gγ9 which robustly translocates to the GA remarkably affects ERK1/2 activation by OR51E2, whereas KO of Gγ3 which does not effectively translocate has no clear effect. In addition, treatment with gallein to inhibit Gβγ function blocks ERK1/2 activation by OR51E2. Therefore, ERK1/2 activation by OR51E2 is most likely mediated through Gβγ dimers, particularly their translocation to the GA. 2) PI3Kγ is a well-characterized signaling molecule acting downstream of Gβγ dimers. Our data have shown that KO of its catalytic subunit p110γ and pharmacological inhibition of its activation abolish ERK1/2 activation by β-ionone, indicative of an essential role played by PI3Kγ. 3) The fact that ARF1 KO suppresses ERK1/2 activation by OR51E2 demonstrates that, similar to PI3Kγ, ARF1 is also an essential element in ERK1/2 activation by OR51E2. 4) Consistent with the role of GA-translocating Gβγ, it is the GA-localized ARF1GEFs, but not the PM-localized ARF1GEFs, which activate ARF1, leading to ERK1/2 activation. Altogether, these data demonstrate a signaling pathway to mediate ERK1/2 activation by OR51E2 in which Gβγ translocation to the GA induces the activation of PI3Kγ and ARF1, and the GA provides a spatial platform in prostate cancer cells.

It is interesting to note that pharmacological inhibition of Gβγ or PI3Kγ attenuates invasiveness or metastatic spread of prostate cancer cells in response to β-ionone stimulation (Sanz et al., 2014; Sanz et al., 2017b), suggesting an important role of Gβγ and PI3Kγ in mediating the function of OR51E2 in prostate cancer cells. Our data presented here have clearly revealed that Gβγ, PI3Kγ and ARF1 mediate ERK1/2 activation by OR51E2 in prostate cancer cells, which are highly consistent with our previous studies on CXCR4-medited ERK1/2 activation (Khater et al., 2021a; Khater et al., 2021b). In addition, genetic depletion and pharmacological inhibition of these three signaling molecules also affect ERK1/2 activation by α2-ARs (Khater et al., 2021b). These data demonstrate that after GPCR activation, Gβγ translocation to the GA which subsequently activates PI3Kγ and ARF1 may provide a common pathway by which multiple GPCRs activate the MAPKs ERK1/2 in prostate cancer cells.

Similar to RTKs, GPCRs play a crucial role in the initiation and progression of many different cancer types and huge efforts are currently underway to develop new GPCR-based drugs for cancer (Dorsam and Gutkind, 2007; Lappano and Maggiolini, 2011). A number of GPCRs, including OR51E2 and CXCR4 (Taichman et al., 2002; Xu et al., 2006), are over-expressed in prostate cancer patients and regulate prostate cancer cell growth, migration and invasion, and prostate cancer progression. These receptors, together with enhanced expression of other signaling molecules, particularly Gγ9 (El-Haibi et al., 2013) and ARF1 (Davis et al., 2016), may partially contribute to hyper-activation of oncogenic MAPK pathway in prostate cancer patients. It is worth noting that inhibition of Gβγ suppresses prostate cancer cell growth and tumor formation (Bookout et al., 2003; Paudyal et al., 2017). These data implicate that enhanced GPCR signaling may represent crucial mechanisms responsible for the hyper-activation of the MAPK pathway in prostate cancer.

In summary, we have demonstrated that ectopically expressed OR51E2 is a potent activator of the MAPKs ERK1/2 in prostate cancer cells and that the function of OR51E2 in activating ERK1/2 is mediated through a specific GA-localized Gβγ-PI3Kγ-ARF1 pathway. Our data provide important insights into the understanding of hyper-activated oncogenic MAPK pathway in prostate cancer.

## Data Availability

The raw data supporting the conclusions of this article will be made available by the authors, without undue reservation.

## References

[B1] Aisenberg W. H. Huang J. Zhu W. Rajkumar P. Cruz R. Santhanam L. (2016). Defining an olfactory receptor function in airway smooth muscle cells. Sci. Rep. 6, 38231. 10.1038/srep38231 27905542 PMC5131280

[B2] Akgoz M. Kalyanaraman V. Gautam N. (2006). G protein betagamma complex translocation from plasma membrane to Golgi complex is influenced by receptor gamma subunit interaction. Cell. Signal. 18, 1758–1768. 10.1016/j.cellsig.2006.01.016 16517125 PMC2230546

[B3] Akgoz M. Kalyanaraman V. Gautam N. (2004). Receptor-mediated reversible translocation of the G protein betagamma complex from the plasma membrane to the Golgi complex. J. Biol. Chem. 279, 51541–51544. 10.1074/jbc.M410639200 15448129

[B4] Bookout A. L. Finney A. E. Guo R. Peppel K. Koch W. J. Daaka Y. (2003). Targeting Gbetagamma signaling to inhibit prostate tumor formation and growth. J. Biol. Chem. 278, 37569–37573. 10.1074/jbc.M306276200 12869546

[B5] Chisari M. Saini D. K. Cho J. H. Kalyanaraman V. Gautam N. (2009). G protein subunit dissociation and translocation regulate cellular response to receptor stimulation. PLoS One 4, e7797. 10.1371/journal.pone.0007797 19936219 PMC2777387

[B6] Crespo P. Xu N. Simonds W. F. Gutkind J. S. (1994). Ras-dependent activation of MAP kinase pathway mediated by G-protein beta gamma subunits. Nature 369, 418–420. 10.1038/369418a0 8196770

[B7] Davis J. E. Xie X. Guo J. Huang W. Chu W. M. Huang S. (2016). ARF1 promotes prostate tumorigenesis via targeting oncogenic MAPK signaling. Oncotarget 7, 39834–39845. 10.18632/oncotarget.9405 27213581 PMC5129974

[B8] Dell'Angelica E. C. Puertollano R. Mullins C. Aguilar R. C. Vargas J. D. Hartnell L. M. (2000). GGAs: A family of ADP ribosylation factor-binding proteins related to adaptors and associated with the Golgi complex. J. Cell. Biol. 149, 81–94. 10.1083/jcb.149.1.81 10747089 PMC2175099

[B9] Donaldson J. G. Jackson C. L. (2011). ARF family G proteins and their regulators: Roles in membrane transport, development and disease. Nat. Rev. Mol. Cell. Biol. 12, 362–375. 10.1038/nrm3117 21587297 PMC3245550

[B10] Dong C. Li C. Wu G. (2011). Regulation of α(2B)-adrenergic receptor-mediated extracellular signal-regulated kinase 1/2 (ERK1/2) activation by ADP-ribosylation factor 1. J. Biol. Chem. 286, 43361–43369. 10.1074/jbc.M111.267286 22025613 PMC3234816

[B11] Dong C. Zhang X. Zhou F. Dou H. Duvernay M. T. Zhang P. (2010). ADP-ribosylation factors modulate the cell surface transport of G protein-coupled receptors. J. Pharmacol. Exp. Ther. 333, 174–183. 10.1124/jpet.109.161489 20093398 PMC2846028

[B12] Dorsam R. T. Gutkind J. S. (2007). G-protein-coupled receptors and cancer. Nat. Rev. Cancer 7, 79–94. 10.1038/nrc2069 17251915

[B13] El-Haibi C. P. Sharma P. Singh R. Gupta P. Taub D. D. Singh S. (2013). Differential G protein subunit expression by prostate cancer cells and their interaction with CXCR5. Mol. Cancer 12, 64. 10.1186/1476-4598-12-64 23773523 PMC3720210

[B14] Gelis L. Jovancevic N. Veitinger S. Mandal B. Arndt H. D. Neuhaus E. M. (2016). Functional characterization of the odorant receptor 51E2 in human melanocytes. J. Biol. Chem. 291, 17772–17786. 10.1074/jbc.M116.734517 27226631 PMC5016170

[B15] Gioeli D. Mandell J. W. Petroni G. R. Frierson H. F. Jr. Weber M. J. (1999). Activation of mitogen-activated protein kinase associated with prostate cancer progression. Cancer Res. 59, 279–284. 9927031

[B16] Imada K. Shiota M. Kohashi K. Kuroiwa K. Song Y. Sugimoto M. (2013). Mutual regulation between Raf/MEK/ERK signaling and Y-box-binding protein-1 promotes prostate cancer progression. Clin. Cancer Res. 19, 4638–4650. 10.1158/1078-0432.CCR-12-3705 23838318

[B17] Irannejad R. Wedegaertner P. B. (2010). Regulation of constitutive cargo transport from the trans-Golgi network to plasma membrane by Golgi-localized G protein betagamma subunits. J. Biol. Chem. 285, 32393–32404. 10.1074/jbc.M110.154963 20720014 PMC2952241

[B18] Jamora C. Yamanouye N. Van Lint J. Laudenslager J. Vandenheede J. R. Faulkner D. J. (1999). Gbetagamma-mediated regulation of Golgi organization is through the direct activation of protein kinase D. Cell. 98, 59–68. 10.1016/S0092-8674(00)80606-6 10412981

[B19] Jensen D. D. Zhao P. Jimenez-Vargas N. N. Lieu T. Gerges M. Yeatman H. R. (2016). Protein kinase D and Gβγ subunits mediate agonist-evoked translocation of protease-activated receptor-2 from the Golgi apparatus to the plasma membrane. J. Biol. Chem. 291, 11285–11299. 10.1074/jbc.M115.710681 27030010 PMC4900274

[B20] Jovancevic N. Khalfaoui S. Weinrich M. Weidinger D. Simon A. Kalbe B. (2017). Odorant receptor 51E2 agonist beta-ionone regulates RPE cell migration and proliferation. Front. Physiol. 8, 888. 10.3389/fphys.2017.00888 29249973 PMC5714887

[B21] Khan S. M. Sleno R. Gora S. Zylbergold P. Laverdure J. P. Labbe J. C. (2013). The expanding roles of Gβγ subunits in G protein-coupled receptor signaling and drug action. Pharmacol. Rev. 65, 545–577. 10.1124/pr.111.005603 23406670

[B22] Khater M. Bryant C. N. Wu G. (2021). Gβγ translocation to the Golgi apparatus activates ARF1 to spatiotemporally regulate G protein-coupled receptor signaling to MAPK. J. Biol. Chem. 296, 100805. 10.1016/j.jbc.2021.100805 34022220 PMC8215300

[B23] Khater M. Wei Z. Xu X. Huang W. Lokeshwar B. L. Lambert N. A. (2021). G protein βγ translocation to the Golgi apparatus activates MAPK via p110γ-p101 heterodimers. J. Biol. Chem. 296, 100325. 10.1016/j.jbc.2021.100325 33493514 PMC7949113

[B24] Klayman L. M. Wedegaertner P. B. (2017). Inducible inhibition of Gβγ reveals localization-dependent functions at the plasma membrane and Golgi. J. Biol. Chem. 292, 1773–1784. 10.1074/jbc.M116.750430 27994056 PMC5290951

[B25] Koch W. J. Hawes B. E. Allen L. F. Lefkowitz R. J. (1994). Direct evidence that Gi-coupled receptor stimulation of mitogen-activated protein kinase is mediated by G beta gamma activation of p21ras. Proc. Natl. Acad. Sci. U. S. A. 91, 12706–12710. 10.1073/pnas.91.26.12706 7809106 PMC45508

[B26] Lappano R. Maggiolini M. (2011). G protein-coupled receptors: Novel targets for drug discovery in cancer. Nat. Rev. Drug Discov. 10, 47–60. 10.1038/nrd3320 21193867

[B27] Madukwe J. C. Garland-Kuntz E. E. Lyon A. M. Smrcka A. V. (2018). G protein βγ subunits directly interact with and activate phospholipase Cϵ. J. Biol. Chem. 293, 6387–6397. 10.1074/jbc.RA118.002354 29535186 PMC5925795

[B28] Malik S. deRubio R. G. Trembley M. Irannejad R. Wedegaertner P. B. Smrcka A. V. (2015). G protein βγ subunits regulate cardiomyocyte hypertrophy through a perinuclear Golgi phosphatidylinositol 4-phosphate hydrolysis pathway. Mol. Biol. Cell. 26, 1188–1198. 10.1091/mbc.E14-10-1476 25609085 PMC4357516

[B29] Mitchell R. McCulloch D. Lutz E. Johnson M. MacKenzie C. Fennell M. (1998). Rhodopsin-family receptors associate with small G proteins to activate phospholipase D. Nature 392, 411–414. 10.1038/32937 9537328

[B30] Mitchell R. Robertson D. N. Holland P. J. Collins D. Lutz E. M. Johnson M. S. (2003). ADP-ribosylation factor-dependent phospholipase D activation by the M3 muscarinic receptor. J. Biol. Chem. 278, 33818–33830. 10.1074/jbc.M305825200 12799371

[B31] Neuhaus E. M. Zhang W. Gelis L. Deng Y. Noldus J. Hatt H. (2009). Activation of an olfactory receptor inhibits proliferation of prostate cancer cells. J. Biol. Chem. 284, 16218–16225. 10.1074/jbc.M109.012096 19389702 PMC2713531

[B32] O'Neill P. R. Karunarathne W. K. Kalyanaraman V. Silvius J. R. Gautam N. (2012). G-protein signaling leverages subunit-dependent membrane affinity to differentially control βγ translocation to intracellular membranes. Proc. Natl. Acad. Sci. U. S. A. 109, E3568–E3577. 10.1073/pnas.1205345109 23213235 PMC3529095

[B33] Paudyal P. Xie Q. Vaddi P. K. Henry M. D. Chen S. (2017). Inhibiting G protein βγ signaling blocks prostate cancer progression and enhances the efficacy of paclitaxel. Oncotarget 8, 36067–36081. 10.18632/oncotarget.16428 28415604 PMC5482639

[B34] Pronin A. Slepak V. (2021). Ectopically expressed olfactory receptors OR51E1 and OR51E2 suppress proliferation and promote cell death in a prostate cancer cell line. J. Biol. Chem. 296, 100475. 10.1016/j.jbc.2021.100475 33640452 PMC8024707

[B35] Rajanala K. Klayman L. M. Wedegaertner P. B. (2021). Gβγ regulates mitotic Golgi fragmentation and G2/M cell cycle progression. Mol. Biol. Cell. 32, br2. 10.1091/mbc.E21-04-0175 34260268 PMC8684744

[B36] Rigau M. Morote J. Mir M. C. Ballesteros C. Ortega I. Sanchez A. (2010). PSGR and PCA3 as biomarkers for the detection of prostate cancer in urine. Prostate 70, 1760–1767. 10.1002/pros.21211 20672322

[B37] Roberts P. J. Der C. J. (2007). Targeting the Raf-MEK-ERK mitogen-activated protein kinase cascade for the treatment of cancer. Oncogene 26, 3291–3310. 10.1038/sj.onc.1210422 17496923

[B38] Rodriguez M. Luo W. Weng J. Zeng L. Yi Z. Siwko S. (2014). PSGR promotes prostatic intraepithelial neoplasia and prostate cancer xenograft growth through NF-κB. Oncogenesis 3, e114. 10.1038/oncsis.2014.29 25111863 PMC5189964

[B39] Rodriguez M. Siwko S. Zeng L. Li J. Yi Z. Liu M. (2016). Prostate-specific G-protein-coupled receptor collaborates with loss of PTEN to promote prostate cancer progression. Oncogene 35, 1153–1162. 10.1038/onc.2015.170 26028029 PMC4683109

[B40] Saini D. K. Kalyanaraman V. Chisari M. Gautam N. (2007). A family of G protein βγ subunits translocate reversibly from the plasma membrane to endomembranes on receptor activation. J. Biol. Chem. 282, 24099–24108. 10.1074/jbc.M701191200 17581822 PMC2238721

[B41] Saini D. K. Karunarathne W. K. Angaswamy N. Saini D. Cho J. H. Kalyanaraman V. (2010). Regulation of Golgi structure and secretion by receptor-induced G protein βγ complex translocation. Proc. Natl. Acad. Sci. U. S. A. 107, 11417–11422. 10.1073/pnas.1003042107 20534534 PMC2895111

[B42] Sanz G. Leray I. Dewaele A. Sobilo J. Lerondel S. Bouet S. (2014). Promotion of cancer cell invasiveness and metastasis emergence caused by olfactory receptor stimulation. PLoS One 9, e85110. 10.1371/journal.pone.0085110 24416348 PMC3885679

[B43] Sanz G. Leray I. Grebert D. Antoine S. Acquistapace A. Muscat A. (2017). Structurally related odorant ligands of the olfactory receptor OR51E2 differentially promote metastasis emergence and tumor growth. Oncotarget. 8, 4330–4341. 10.18632/oncotarget.13836 28032594 PMC5354835

[B44] Sanz G. Leray I. Muscat A. Acquistapace A. Cui T. Riviere J. (2017). Gallein, a Gβγ subunit signalling inhibitor, inhibits metastatic spread of tumour cells expressing OR51E2 and exposed to its odorant ligand. BMC Res. Notes 10, 541. 10.1186/s13104-017-2879-z 29084601 PMC5663063

[B45] Schutzman J. L. Martin G. R. (2012). Sprouty genes function in suppression of prostate tumorigenesis. Proc. Natl. Acad. Sci. U. S. A. 109, 20023–20028. 10.1073/pnas.1217204109 23150596 PMC3523874

[B46] Senarath K. Payton J. L. Kankanamge D. Siripurapu P. Tennakoon M. Karunarathne A. (2018). Gγ identity dictates efficacy of Gβγ signaling and macrophage migration. J. Biol. Chem. 293, 2974–2989. 10.1074/jbc.RA117.000872 29317505 PMC5827438

[B47] Shepard B. D. Natarajan N. Protzko R. J. Acres O. W. Pluznick J. L. (2013). A cleavable N-terminal signal peptide promotes widespread olfactory receptor surface expression in HEK293T cells. PLoS One 8, e68758. 10.1371/journal.pone.0068758 23840901 PMC3698168

[B48] Spehr J. Gelis L. Osterloh M. Oberland S. Hatt H. Spehr M. (2011). G protein-coupled receptor signaling via Src kinase induces endogenous human transient receptor potential vanilloid type 6 (TRPV6) channel activation. J. Biol. Chem. 286, 13184–13192. 10.1074/jbc.M110.183525 21349844 PMC3075665

[B49] Taichman R. S. Cooper C. Keller E. T. Pienta K. J. Taichman N. S. McCauley L. K. (2002). Use of the stromal cell-derived factor-1/CXCR4 pathway in prostate cancer metastasis to bone. Cancer Res. 62, 1832–1837. 11912162

[B50] Uzgare A. R. Kaplan P. J. Greenberg N. M. (2003). Differential expression and/or activation of P38MAPK, erk1/2, and jnk during the initiation and progression of prostate cancer. Prostate 55, 128–139. 10.1002/pros.10212 12661038

[B51] Wang J. Weng J. Cai Y. Penland R. Liu M. Ittmann M. (2006). The prostate-specific G-protein coupled receptors PSGR and PSGR2 are prostate cancer biomarkers that are complementary to alpha-methylacyl-CoA racemase. Prostate 66, 847–857. 10.1002/pros.20389 16491480

[B52] Weber M. J. Gioeli D. (2004). Ras signaling in prostate cancer progression. J. Cell. Biochem. 91, 13–25. 10.1002/jcb.10683 14689577

[B53] Wei Z. Xu X. Fang Y. Khater M. Naughton S. X. Hu G. (2021). Rab43 GTPase directs post-synaptic trafficking and neuron-specific sorting of G protein-coupled receptors. J. Biol. Chem. 296, 100517. 10.1016/j.jbc.2021.100517 33676895 PMC8050390

[B54] Wei Z. Zhang M. Li C. Huang W. Fan Y. Guo J. (2019). Specific TBC domain-containing proteins control the ER-golgi-plasma membrane trafficking of GPCRs. Cell. Rep. 28, 554–566. e554. 10.1016/j.celrep.2019.05.033 31291588 PMC6639060

[B55] Weng J. Wang J. Cai Y. Stafford L. J. Mitchell D. Ittmann M. (2005). Increased expression of prostate-specific G-protein-coupled receptor in human prostate intraepithelial neoplasia and prostate cancers. Int. J. Cancer 113, 811–818. 10.1002/ijc.20635 15499628

[B56] Wu G. Benovic J. L. Hildebrandt J. D. Lanier S. M. (1998). Receptor docking sites for G-protein betagamma subunits. Implications for signal regulation. J. Biol. Chem. 273, 7197–7200. 10.1074/jbc.273.13.7197 9516410

[B57] Wu G. Bogatkevich G. S. Mukhin Y. V. Benovic J. L. Hildebrandt J. D. Lanier S. M. (2000). Identification of Gbetagamma binding sites in the third intracellular loop of the M(3)-muscarinic receptor and their role in receptor regulation. J. Biol. Chem. 275, 9026–9034. 10.1074/jbc.275.12.9026 10722752

[B58] Wu G. Zhao G. He Y. (2003). Distinct pathways for the trafficking of angiotensin II and adrenergic receptors from the endoplasmic reticulum to the cell surface: Rab1-independent transport of a G protein-coupled receptor. J. Biol. Chem. 278, 47062–47069. 10.1074/jbc.M305707200 12970354

[B59] Xia C. Ma W. Wang F. Hua S. Liu M. (2001). Identification of a prostate-specific G-protein coupled receptor in prostate cancer. Oncogene 20, 5903–5907. 10.1038/sj.onc.1204803 11593396

[B60] Xie H. Liu T. Chen J. Yang Z. Xu S. Fan Y. (2019). Activation of PSGR with beta-ionone suppresses prostate cancer progression by blocking androgen receptor nuclear translocation. Cancer Lett. 453, 193–205. 10.1016/j.canlet.2019.03.044 30928381

[B61] Xu L. L. Sun C. Petrovics G. Makarem M. Furusato B. Zhang W. (2006). Quantitative expression profile of PSGR in prostate cancer. Prostate Cancer Prostatic Dis. 9, 56–61. 10.1038/sj.pcan.4500836 16231015

[B62] Xu X. Wei Z. Wu G. (2022). Specific motifs mediate post-synaptic and surface transport of G protein-coupled receptors. iScience 25, 103643. 10.1016/j.isci.2021.103643 35024582 PMC8728401

[B63] Zhou F. Dong C. Davis J. E. Wu W. H. Surrao K. Wu G. (2015). The mechanism and function of mitogen-activated protein kinase activation by ARF1. Cell. Signal. 27, 2035–2044. 10.1016/j.cellsig.2015.06.007 26169956 PMC4739547

